# Electrochemical Investigation of Adsorption of Single‐Wall Carbon Nanotubes at a Liquid/Liquid Interface

**DOI:** 10.1002/open.201600136

**Published:** 2016-12-13

**Authors:** Aminu K. Rabiu, Peter S. Toth, Andrew N. J. Rodgers, Robert A. W. Dryfe

**Affiliations:** ^1^ School of ChemistryUniversity of ManchesterOxford RoadManchesterM13 9PLUK

**Keywords:** adsorption, carbon nanotubes, electrochemistry, interface assembly, ion transfer

## Abstract

There is much interest in understanding the interfacial properties of carbon nanotubes, particularly at water/oil interfaces. Here, the adsorption of single‐wall carbon nanotubes (SWCNTs) at the water/1,2‐dichloroethane (DCE) interface, and the subsequent investigation of the influence of the adsorbed nanotube layer on interfacial ion transfer, is studied by using the voltammetric transfer of tetramethylammonium (TMA^+^) and hexafluorophosphate (PF_6_^−^) as probe ions. The presence of the interfacial SWCNT layer significantly suppresses the transfer of both ions across the interface, with a greater degree of selectivity towards the PF_6_^−^ ion. This effect was attributed both to the partial blocking of the interface by the SWCNTs and to the potential dependant adsorption of background electrolyte ions on the surface of the SWCNTs, as confirmed by X‐ray photoelectron spectroscopy, which is caused by an electrostatic interaction between the interfacial SWCNTs and the transferring ion.

## 1 Introduction

Carbon nanotubes (CNTs) have attracted a great deal of attention owing to their interesting optical, mechanical, and electrical properties.^1^ Their potential application in a variety of areas such as nanoelectronics,^2^ field effect transistors,^3^ electrochemical and sensor devices,1a, 4 and as catalyst support^5^ has been demonstrated. However, for most applications in nanoscience and technology, the processing and subsequent formation of stable assemblies of these nanostructures is highly important.^6^ Liquid/liquid interfaces, particularly the oil/water interface, have become increasingly popular for the assembly of a wide range of nanostructures, such as metal nanoparticles and two dimensional (2D) semiconducting nanomaterials.^7^

The self‐assembly of both single‐wall carbon nanotubes (SWCNTs) and multi‐walled carbon nanotube (MWCNTs) at a variety of liquid/liquid interfaces has also been explored greatly as an alternative method to generate functional CNT films.^8^ Typically, the material to be assembled at the interface (i.e. CNTs, in this case) is suspended in one of the bulk liquid phases. The suspension is then contacted with the second liquid phase and assembly is subsequently induced by mechanical agitation or addition of an inducing solvent.^9^ Although the majority of these studies—in the case of CNTs—focus more on the assembly process, few have investigated the properties of the CNT layers/films in situ at these interfaces. For example, Matsui et al.8c fabricated ultrathin films, or 2D layers, of SWCNTs at the water/*n*‐hexane interface and characterized their optical and electrical properties ex situ, after transfer of the films onto a silicon wafer. The use of SWCNTs to transport enzymes from a bulk aqueous phase to a water/organic interface, and the subsequent characterization of the biocatalytic activity of the resulting SWCNT–enzyme interfacial layer has been examined, with an enhancement in the rate of biotransformation observed with the interfacial layer.^10^ This was interpreted in terms of the high intrinsic surface area provided by the SWCNTs and the absence of intraparticle diffusion limitations. Zhang et al.^11^ obtained a flexible thin film of imidazolium‐functionalized SWCNTs (Im‐SWCNTs) at a non‐polarized water/chloroform interface and attempted electrochemical characterization of the resultant interfacial layer by using scanning electrochemical microscopy (SECM). With only the oxidized form of the redox species [Ru(NH_3_)_6_^3+^] present in the aqueous phase, it was shown that, at the “bare” water/chloroform interface, a negative feedback current was generated as the tip approached the interface, owing to the interface acting as an insulator; whereas, in the presence of an Im‐SWCNTs interfacial layer, a positive feedback current was generated at the tip, indicating that the Im‐SWCNTs film was electroactive. However, as there was no redox species in the chloroform phase, no charge‐transfer reaction occurred between the two immiscible liquids.

In the presence of appropriate electrolytes dissolved in each liquid phase, the liquid/liquid interface is referred to as the interface between two immiscible electrolyte solutions (ITIES). This special class of liquid/liquid interface can be polarized by the application of an external electric field, thus allowing both ion‐ and electron‐transfer reactions to be readily studied by using electrochemical methods.^12^ The modification of the ITIES with adsorbed solids has been shown to be a viable means for studying the properties of interfacially adsorbed materials, such as membrane porosity^13^ and catalytic activity of metal nanoparticles.^14^

Recent studies in our laboratory have utilized this approach to probe the electrochemical properties of graphitic carbon nanostructures (CNTs and few‐layer graphene) adsorbed at the ITIES. It was shown that interfacially assembled SWCNT/graphene layers serve as electron mediators, aiding heterogeneous electron transfer between aqueous and organic redox couples, which remain isolated in their respective phases.^15^ This was utilized to functionalize interfacial SWCNT and graphene layers with metal nanoparticles by reducing aqueous metal salts using an organic electron donor,15a, 16 and a conducting polymer poly(pyrrole),^17^ through oxidation of the pyrrole monomer dissolved in the organic phase by an aqueous oxidizing agent. Similarly, the electron‐transfer‐mediating properties of pristine liquid‐phase exfoliated graphene at the water/organic interface were found to result in a catalytic effect on the heterogeneous oxygen reduction reaction.^18^ Furthermore, the electrochemical doping of the interfacial SWCNTs was investigated by using in situ Raman spectroelectrochemistry.15b

The objective of the current work is to investigate the electrical properties of SWCNTs adsorbed at the water/DCE interface through analysis of their effect on the kinetics of ion transfer across the interface. The permeability of the films formed at different SWCNTs concentration by the ionic species is also described.

## 2 Results and Discussion

### 2.1 SWCNT Adsorption at Water/DCE Interface

**Figure 1:**
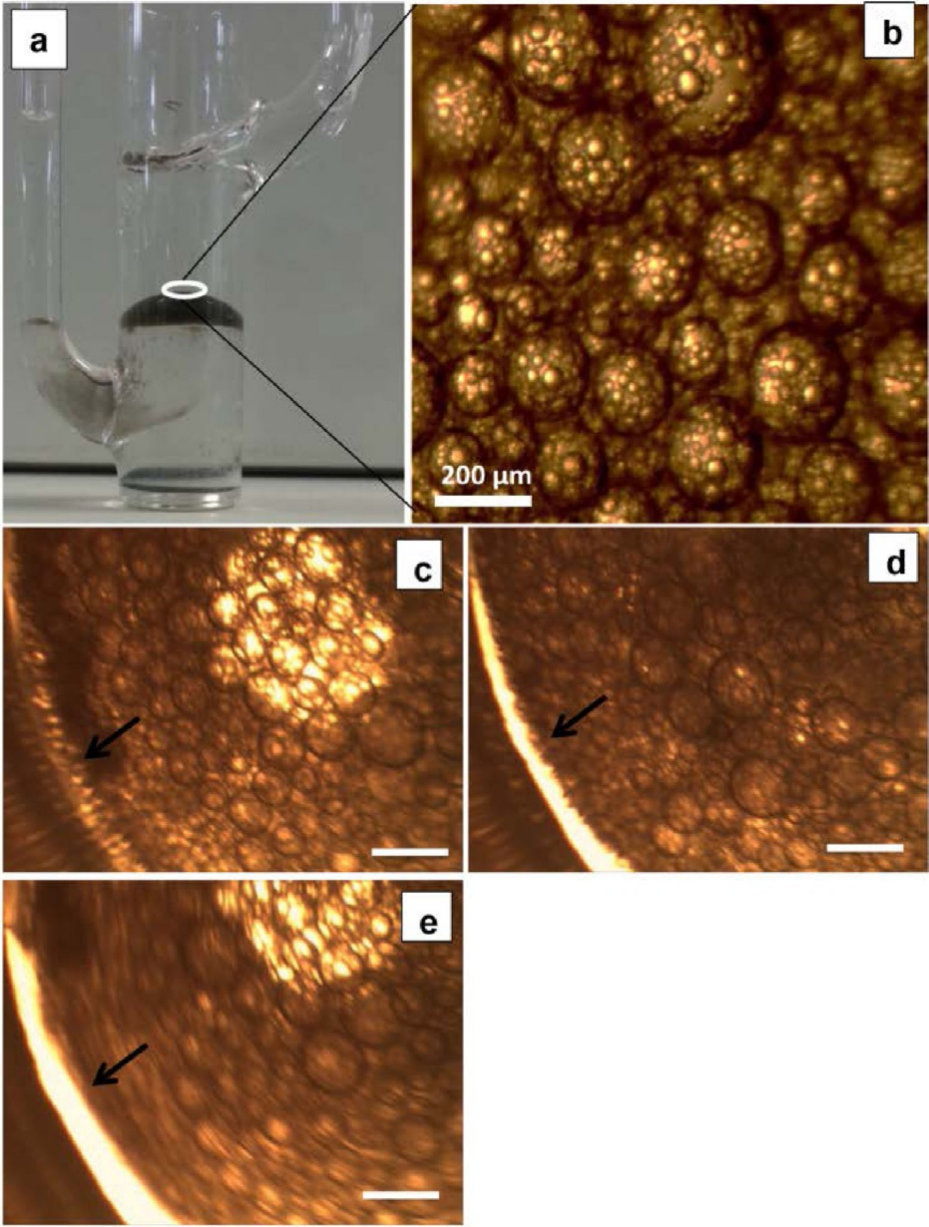
a) Photograph of the electrochemical cell, showing an example of a SWCNT film at the liquid/liquid interface 12 h after sonication. *C*_SWCNT_ used for film preparation was 6 mg L^−1^. In situ optical microscopy images of SWCNT interfacial films taken b) in the absence of an applied potential (*C*_SWCNT_ was 2 mg L^−1^) and c–e) at an interfacial potential of −0.24 V (c), +0.06 V (d), or +0.46 V (e); *C*_SWCNT_ was 1 mg L^−1^ [scale bars in (c)–(e) represent 150 μm]. The arrow indicates the cell wall.

Interfacial SWCNT layers were formed following a 10 min bath sonication of cells containing a DCE dispersion of SWCNTs and an aqueous phase solution. The SWCNT film located between the bulk phases was visible a few minutes after sonication. However, owing to some emulsification of both the water/DCE interface and the bulk liquid phases, caused by the sonication, the cells were left to stand for 12 h to allow the emulsion droplets in the bulk phases to coalesce before carrying out any electrochemical measurements. Figure 1 a shows a typical cell 12 h after sonication.

The resultant interfacial SWCNT film was characterized in situ by using optical microscopy and ex situ by using scanning electron microscopy (SEM). Figure 1 b shows an optical micrograph of a SWCNT film obtained by using a DCE dispersion concentration of 3 mg L^−1^. As can be seen, the interfacial film was composed of multiple emulsified droplets stabilized by SWCNTs. These droplets were observed to be stable for up to 7 days when left undisturbed. Longer time stability was not studied here. The droplets were also found to be stable when the water/DCE interface was polarized, as exemplified by the microscopy images in Figure 1 c–e. The images showing the morphology of the interfacial layer at different applied potential differences across the water/DCE interface (Δ*φ*) were captured during a cyclic voltammetry experiment, where Δ*φ* was swept from −0.24 to +0.46 V. The only effect observed was the movement of the whole interfacial film towards one side of the interface on positive polarization (indicated by the arrows, showing that as Δ*φ* was swept from −0.24 to +0.46 V, the space between the film and the wall of the glass increases) and vice‐versa on reverse polarization. This movement of the SWCNT film may be connected with the movement of individual interfacial SWCNTs, as previously highlighted by the Girault group.5a At extreme positive interfacial potential difference (Δ*φ≥*+0.46 V), corresponding to background ion transfer, the interfacial film rotates clockwise on positive scan and anticlockwise on reverse (negative) scan. Figure 1 e was taken during this rotation, which is the reason it appear like slightly out of focus.

**Figure 2:**
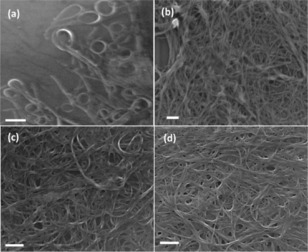
Ex situ SEM images showing the morphologies of the SWCNTs films formed at the liquid/liquid interface using a *C*_SWCNT_ of a) 1 mg L^−1^, b) 6 mg L^−1^, c) 12 mg L^−1^ L, and d) 18 mg L^−1^.

Figure 2 shows ex situ SEM images of interfacial SWCNTs films prepared at different SWCNT dispersion concentrations. The interfacial films were carefully transferred onto a Si/SiO_2_ substrate prior to SEM measurement. As can be seen, the interfacial preparation method resulted in two types of SWCNT film morphologies depending on the initial dispersion concentration: at low SWCNT concentration (1 mg L^−1^), the SWCNTs were predominately bent into rings (Figure 2 a), with only a few straight or partially bent tubes, whereas at the higher SWCNT concentrations of 6, 12, and 18 mg L^−1^, porous interfacial films were formed, composed of random networks of multilayer SWCNTs (Figure 2 b–d). The density of these multilayer films can be seen to increase with increasing nanotube concentration, although the density of the films obtained with 12 and 18 mg L^−1^ CNT concentrations were very similar. The observed concentration‐dependent transition of SWCNT morphologies from rings to straight tubes is similar to the findings of Wang et al. for a water/DCB Pickering emulsion system stabilized by SWCNTs.^19^

### 2.2 Ion Transfer across SWCNT Interfacial Films

**Scheme 1:**
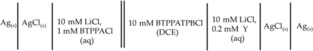
Schematic of the electrochemical cell used in ion transfer studies. Y is either TMA^+^ or PF_6_^−^.

**Figure 3:**
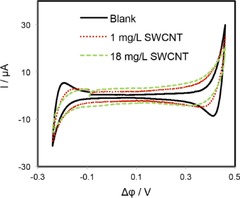
CVs of the supporting electrolytes obtained in the absence (black line) and presence of SWCNTs films prepared at different bulk CNTs concentrations (green and red lines).

The composition of the cell employed for all electrochemical measurements is outlined in Scheme 1. Figure 3 shows the cyclic voltammograms (CVs) obtained in the presence of only the background electrolytes at the bare water/DCE interface and with interfacial SWCNT films prepared from two different bulk SWCNT dispersion concentrations (6 and 18 mg L^−1^). It can be seen that, in the presence of the interfacial SWCNT layers, there was a slight increase in the capacitive current. This can be attributed to an increase in liquid/liquid interfacial roughness, owing to the presence of multiple emulsion droplets formed at the interface when the SWCNTs are adsorbed (Figure 1).^20^ An alternative explanation is to consider the relative capacitances of the “free” and “blocked” parts of the interface; however, such an argument would lead to a decrease in the net capacitance, owing to the low capacitance of carbon nanotubes.^21^ Further increases in the bulk SWCNT concentration resulted in very little increase in the capacitive current. Additionally, the interfacial SWCNT films affected the magnitude and shape of the background electrolyte ion transfer peaks (Li^+^ and Cl^−^), which limit the potential window on the positive and negative ends, respectively. The current magnitudes were reduced and the transfer peaks became broader, indicating that the presence of an interfacial SWCNT film makes the ion transfer more difficult.

### 2.3 TMA^+^ and PF_6_^−^ Ion Transfer

**Figure 4:**
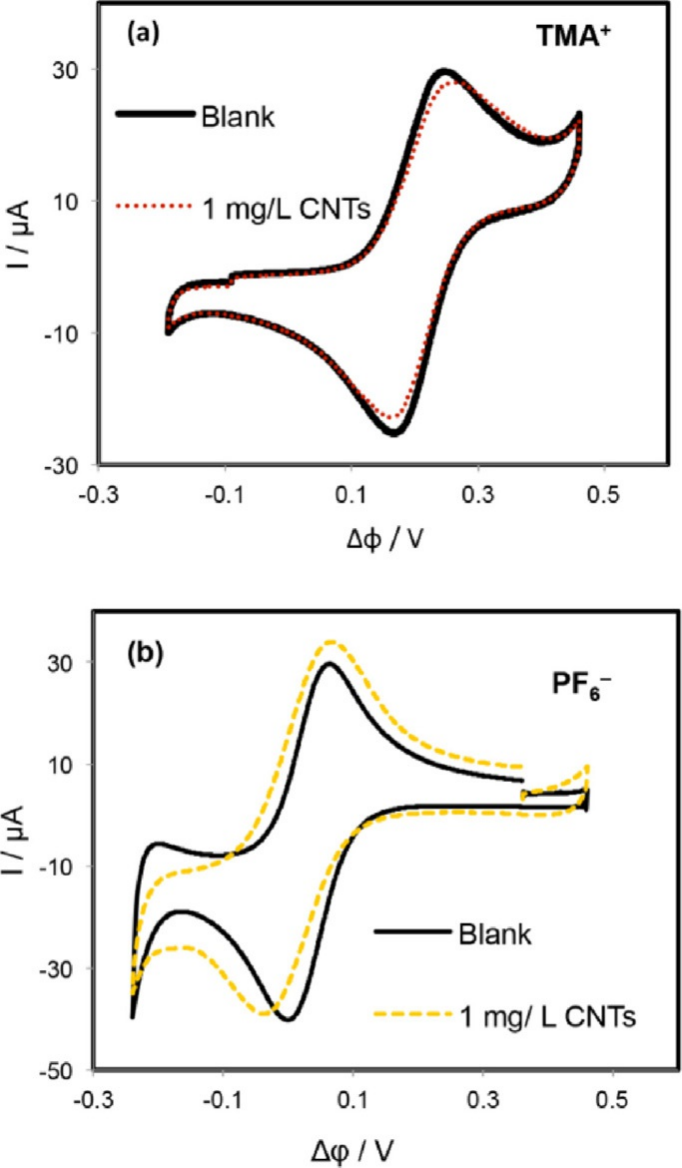
CVs obtained for the transfer of TMA^+^ (a) and PF_6_^−^ (b) in the absence (solid lines) and in the presence (dash lines) of SWCNT films prepared at *C*_SWCNTs_ of 1 mg L^−1^. Scan rate was 50 mV s^−1^.

The blocking effect of SWCNTs interfacial films on ion transfer was further investigated by employing TMA^+^ and PF_6_^−^ as probe ions. First, the transfer of each ion was performed in the absence of SWCNT layers and then repeated in the presence of interfacial SWCNT films of differing thickness. The CVs shown in Figure 4 were obtained for TMA^+^ and PF_6_^−^ ions in the presence of SWCNT films prepared from bulk SWCNT concentrations of 1 mg L^−1^. Also shown in the figure are CVs obtained at the unmodified interface for comparison. It can be seen that the responses of both TMA^+^ and PF_6_^−^ ions were very similar to those obtained in the absence of the interfacial SWCNTs films. There was only a small increase in peak separation (Δ*E*_p_) and a slight reduction in the peak current magnitudes.

**Figure 5:**
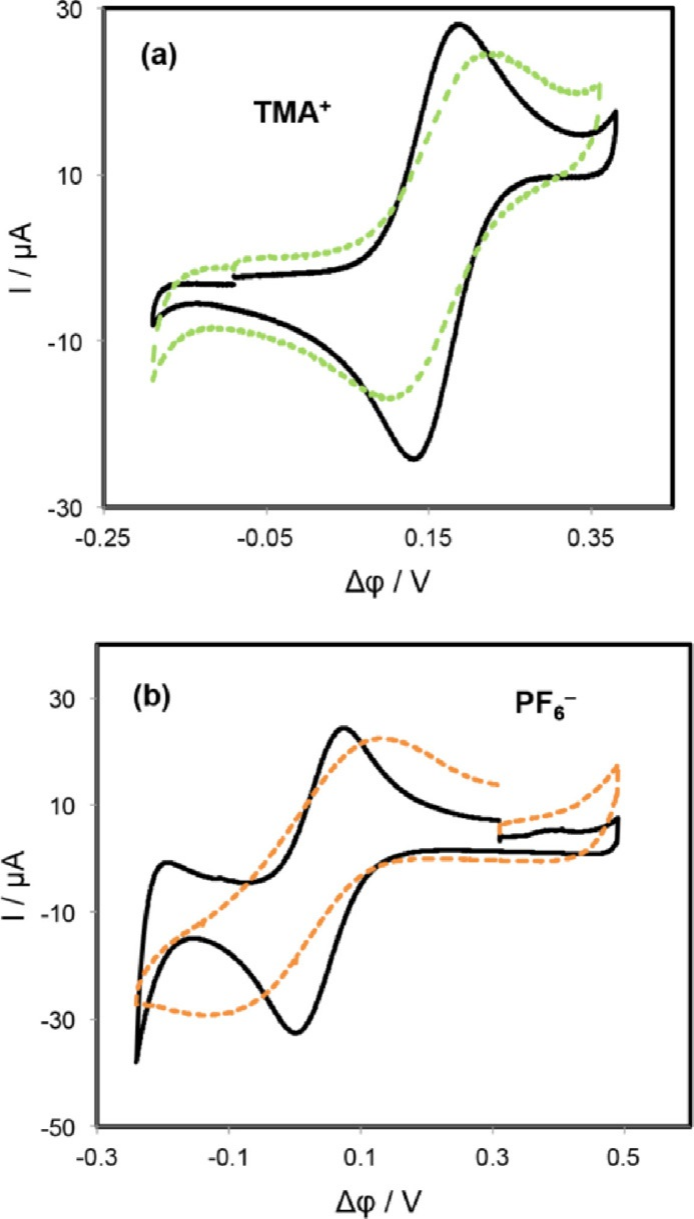
CVs obtained for the transfer of TMA^+^ (a) and PF_6_^−^ (b) in the absence (solid lines) and in the presence (dash lines) of SWCNTs films prepared at a *C*_SWCNTs_ of 6 mg L^−1^. Scan rate was 50 mV s^−1^.

However, when the nanotube dispersion concentration used for the film preparation was increased to 6 mg L^−1^, the response obtained in all cases was significantly altered, as compared to those obtained at the bare interface (Figure 5); both forward and reverse transfer peaks were broadened and shifted away from each other and their magnitudes decreased. This behavior indicates that increasing the SWCNT dispersion concentration leads to a higher interfacial surface coverage, resulting in a greater part of the interface available for ion transfer being blocked by the SWCNTs.

**Figure 6:**
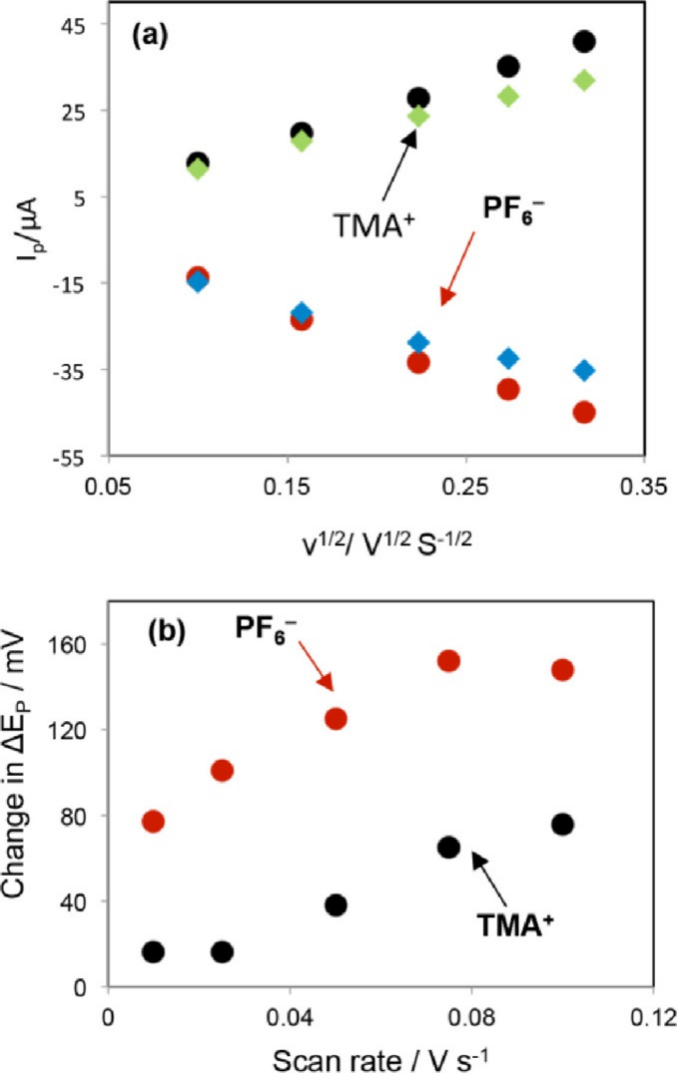
a) Plot of forward transfer peak current for TMA^+^ and PF_6_^−^ ions as a function of *ν* ^1/2^ in the absence (circles) and in the presence (squares) of a SWCNT film prepared by using a *C*_SWCNTs_ of 6 mg L^−1^. b) Change in Δ*E*_p_ (Δ*E*_p_ in the presence of SWCNTs−Δ*E*_p_ in the absence of SWCNTs) as a function of scan rate for each probe ion. *C*_SWCNTs_ used for the assembly was 6 mg L^−1^.

Figure 6 a shows a graph of the dependence of forward peak height (*I*_pf_), measured at bare and at SWCNT‐covered interfaces, against the square root of the scan rate (*ν* ^1/2^) for both TMA^+^ and PF_6_^−^. In each case, *I*_pf_ was linearly related to *ν* ^1/2^ and the decrease in *I*_pf_ was similar for both ions. Using the Randles–Ševčik relation for the data collected from the bare water/DCE interface, the aqueous diffusion coefficient (*D*_w_) of each ion was calculated. *D*_w_ values obtained for TMA^+^ (1.2×10^5^ cm^2^ s^−1^) and PF_6_^−^ (1.4×10^5^ cm^2^ s^−1^) were in agreement with the literature values of 1.2×10^5^ cm^2^ s^−1[22]^ for TMA^+^ and 1.5×10^5^ cm^2^ s^−1[23]^ for PF_6_^−^. Figure 6 b shows the change in Δ*E*_p_ for each ion as a function of scan rate in the presence of SWCNTs. It can be seen that the change in Δ*E*_p_ is greater for PF_6_^−^ compared to TMA^+^, which indicates that the kinetics of the PF_6_^−^ transfer were more inhibited by the interfacial SWCNT film.

To rationalize this observed ion selectivity, the possible adsorption of either the probe ions or the organic background electrolyte ions on the assembled SWCNT film was investigated by using chronoamperometry and XPS. Firstly, potential step experiments were performed for each probe ion transfer. The interfacial potential was stepped from a potential where no ion transfer occurs (−0.1 and +0.25 V for TMA^+^ and PF_6_^−^, respectively) to a potential where ion transfer from the water to organic phase takes place (+0.25 and −0.18 V for TMA^+^ and PF_6_^−^_,_^.^ spectively). The interfacial potential was held at the ion transfer potential for 10 min, after which the SWCNT film was carefully transferred onto a Si/SiO_2_ wafer. The transferred layers were then washed in ethanol, isopropanol, and acetone and dried before subsequent XPS analysis. A control sample was treated in a similar way, with the exception that neither the probe ions nor the supporting electrolyte ions were present and no interfacial potential was applied.

**Figure 7:**
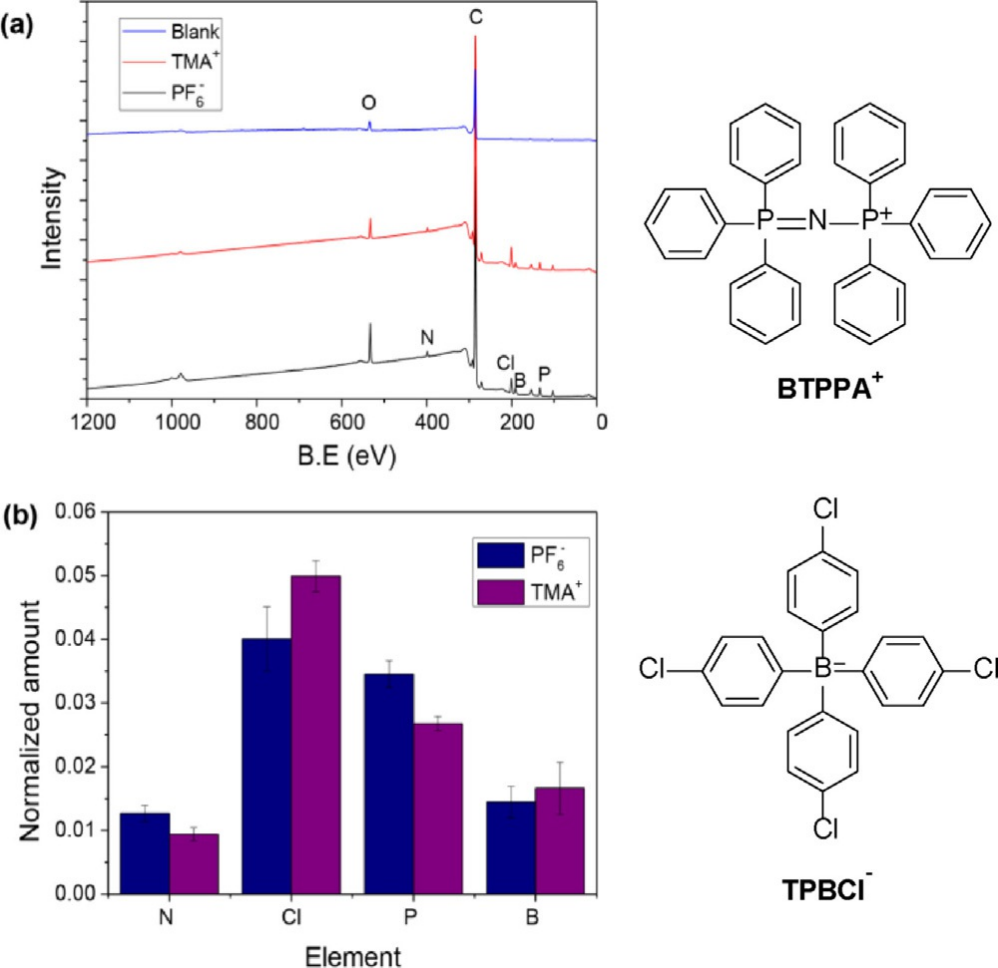
a) XPS survey spectra obtained for the SWCNT layer extracted from the water/DCE interface after the transfer of TMA^+^ and PF_6_^−^ probe ions from water to DCE phase. b) Atomic concentrations of B, N, Cl, and P (normalized to carbon) evaluated from the XPS survey spectra.

Figure 7 a presents XPS spectra of the aforementioned SWCNT films. The spectra show the presence of B, N, Cl, and P in films obtained with either TMA^+^ or PF_6_^−^ present, but not in the control sample. The appearance of signals attributable to B, Cl, and P in the TMA^+^ and PF_6_^−^ samples is indicative of adsorption of the aromatic cation, BTPPA^+^, and anion, TPBCl^−^, of the organic supporting electrolyte on the SWCNT surface, as substantial amounts of these elements could only be reasonably attributed to the supporting electrolyte ions. The absence of a significant signal for fluorine in any sample, and particularly in the sample obtained with PF_6_^−^ present, suggests that the PF_6_^−^ was only weakly adsorbed or not adsorbed at all. The percentage atomic concentrations of B, N, Cl, and P determined from the survey spectra of TMA^+^ and PF_6_^−^ samples were normalized to that of C, and the results are summarized in Figure 7 b. The P/N ratio of approximately 2:1 is consistent with the stoichiometric composition of BTPPA^+^. The absence of additional N in the TMA^+^ sample could suggest that the TMA^+^ ion only weakly adsorbs, or not at all, on the SWCNT surface. Similarly, the B/Cl ratio was found to be close to the 1:4 expected for the TPBCl^−^ anion. The slight excess of B was attributed to overlap of the B 1s and P 2s peaks, which made it difficult to accurately subtract the contribution of the P 2s signal. Nevertheless, the XPS data clearly demonstrate the preferential adsorption of BTPPA^+^ and TPBCl^−^ on the SWCNTs surface over the TMA^+^ and PF_6_^−^ ions, which is plausible considering that both BTPPA^+^ and TPBCl^−^ are charged and could also interact with the SWCNTs through π–π stacking.^24^ The structure of these electrolyte ions are shown in Figure 7. Furthermore, the XPS data presented in Figure 7 b show a potential‐dependent adsorption of the supporting electrolyte ions on the interfacial SWCNTs, as illustrated by the relative intensities of the components obtained for the samples containing TMA^+^ and PF_6_^−^. It can be seen that at the TMA^+^ transfer potential (+0.25 V), the N and P peaks, attributable to BTPPA^+^, are lower in intensity compared to those measured at the PF_6_^−^ transfer potential (−0.18 V), whereas the intensities of the B and Cl peaks from TPBCl^−.^ a lower for the PF_6_^−^ transfer potential than that of TMA^+^. Overall, the XPS data suggest that asymmetric adsorption of the supporting electrolyte ions occurs on the interfacial SWCNTs, introducing a net negative or positive surface charge on the SWCNTs at the TMA^+^ and PF_6_^−^ transfer potentials, respectively, thereby resulting in the retardation of ion transfer across the interface through electrostatic attraction between the transferring ion and the adsorbed supporting electrolyte counter ion. The difference in the extent of charge‐transfer suppression between the two probe ions is associated with the relative positions of the transfer potentials of the probe ions with respect to the potential of zero charge (*PZC*) in the presence of the modified SWCNTs. The fact that the kinetics of PF_6_^−^ ion transfer is affected more than that of the TMA^+^ ion implies that the SWCNT film has a higher charge density at the PF_6_^−^ transfer potential, causing more electrolyte ions to adsorb on its surface and, consequently, increasing the electrostatic attraction between the PF_6_^−^ ion and the adsorbed BTPPA^+^ cation. For the TMA^+^ ion to be less hindered, its transfer potential should be closer to the *PZC* of the system, which results in less attraction between the TMA^+^ and the adsorbed TPBCl^−^ anion.

### 2.4 Kinetics of Ion Transfer

**Figure 8:**
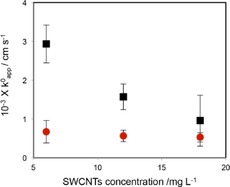
Plot of the apparent rate constant versus the SWCNT concentration used in film preparation. Black squares and red circles denote kinetic data for TMA^+^ and PF_6_^−^, respectively.

The apparent rate constant (*k* ^0^_app_) of TMA^+^ and PF_6_^−^ ion transfer in the presence of SWCNTs films was determined by using the Nicholson method.^25^ Δ*E*_p_ values measured at scan rates higher than 25 mV s^−1^ were used. Figure 8 shows the *k* ^0^_app_ values obtained. Increasing the SWCNT dispersion concentration in the organic phase resulted in a decrease in *k* ^0^_app_ for the TMA^+^ ion, owing to the greater surface coverage by the SWCNTs (Figure 2). This can be explained by invoking Amatore's theory of voltammetry^26^ at a partially blocked electrode if we assume that the SWCNTs have transformed the single continuous interfacial area into a large number of smaller randomly distributed micro‐/nanopores, the size and/or density of which decreases with increased interfacial coverage. According to the theory,^26^ under conditions of total overlap of the diffusion layers, *k* ^0^_app_ is lowered by a factor of (1−*θ*) [Eq. (1)]:

(1) $$k{}^{0}{}_{\mathrm{app}}=k{}_{0}\;\left(1-\theta \right)$$

where *θ* is defined as the fractional area covered by the blocking nanotube film.

The fact that the voltammetric profile of TMA^+^ transfer exhibited a peak‐shaped response rather than a sigmoidal one indicates that an overlapping linear diffusion field was achieved. The interfacial coverage was estimated from the SEM data to be about 77.5, 88.0, and 91.7 % when the SWCNT concentration used in film preparation was 6, 12, and 18 mg L^−1^ respectively. Therefore, applying the (1−*θ*) correction factor gave an average *k*_0_ value of 1.0±0.1×10^−2^ cm s^−1^.

Aside from the slower kinetics displayed by the PF_6_^−^ ion compared to TMA^+^ transfer in the presence of interfacially assembled SWCNTs (Figures 6 b and 8), it is also clear from Figure 8 that the negative probe ion also show a less clear dependence of *k* ^0^_app_ on SWCNT concentration. The ion transfer and the XPS data indicate that there is a potential‐dependent change in surface composition of the nanotubes, which in turn suggests that the nanotubes adsorb on the interface from the organic phase, that is, they constitute part of organic double layer. This effect is then associated with the high surface charge density exhibited by the interfacial SWCNTs at the PF_6_^−^ transfer potential, which leads to the attainment of maximum blockage at the SWCNT concentration of 6 mg L^−1^, as against the TMA^+^ ion.

## 3 Conclusions

In the present study, we have demonstrated the use of ion transfer voltammetry at the liquid/liquid interface to characterize the electrical properties of SWCNTs adsorbed at a water/DCE interface. In the presence of adsorbed SWCNT layers of varying density/thickness, transfer of the positively charged TMA^+^ ion across the interface was found to be less inhibited than the corresponding negative PF_6_^−^ ion. The retardation of ion transfer by the nanotube layer was analyzed by using the theory of voltammetry at partially blocked electrodes, and the selectivity between TMA^+^ and PF_6_^−^ ions was attributed to the potential‐dependent adsorption of the organic supporting electrolyte ions on the interfacial SWCNTs, as indicated by XPS measurements, which caused electrostatic interaction between the transferring ion and the SWCNT surface and, thereby, inhibited the ion transfer.

## Experimental Section

### Materials

Arc discharge SWCNTs (purified, >95 % carbon), 1,2‐dichloroethane (DCE, ≥99.8 %), lithium chloride (LiCl, ≥99 %), tetramethylammonium chloride (TMACl, ≥99 %), sodium hexafluorophosphate (NaPF_6_, 99.99 %), potassium chloride (KCl, 99.8 %) potassium tetrakis (4‐chlorophenyl) borate (KTPBCl, ≥98 %), and bis(triphenylphosporanylidene) ammoniumchloride (BTPPACl, 97 %) were purchased from Sigma Aldrich and used as received. Dibenzo‐18‐crown‐6 (98+%) was a product of Lancaster Synthesis. The bis(triphenylphosporanylidene) ammonium tetrakis(4‐chlorophenyl) borate (BTPPATPCl) used as the organic‐phase electrolyte was prepared as described previously.^18^, ^27^ Ultrapure water (18.2 MΩ cm resistivity, Milli‐Q Direct 8, Merck Millipore, USA) was used for aqueous solutions preparation.

### Methods

SWCNT dispersions in DCE were prepared by sonication. Pristine SWCNTs (22 mg) were placed in a 500 mL flat‐bottom glass bottle containing DCE (100 mL). The contents were bath sonicated for 24 h by using an Elmasonic P70 H sonicator (Elma GmbH & Co. KG) at 37 KHz and 30 % power setting. The as‐prepared dispersion was stable for months. Aliquots of the dispersion were taken, diluted, and used to determine the extinction coefficient (*α*) by using UV/Vis absorption spectroscopy. The value of *α* obtained at 660 nm was 39±0.9×10^2^ mg^−1^ mL m^−1^ and agrees with 41.00 ±0.4×10^2^ mg^−1^ mL m^−1^ reported previously for CVD‐grown SWCNTs dispersed in DCE.^16^ Self‐assembly of the SWCNTs at the water/DCE interface was achieved by following the procedure reported previously in our laboratory.^15^ Briefly, an aliquot of the SWCNT dispersion in DCE was mixed with the organic supporting electrolyte and an equal volume of the aqueous phase was placed on top of this organic phase. Assembly was then induced by a 10 min bath sonication (37 kHz and 40 % power). Cyclic voltammetry and potential step experiments were carried out with an Autolab potentiostat PGSTAT20 (Metrohm‐Autolab) operated in a four‐electrode configuration mode with *IR* compensation applied during all cyclic voltammetry measurements. The applied potential was converted to Galvani potential difference (Δ*φ*) by using the standard ion transfer of TMA^+^ ion (Δw0 *φ*) taken as +160 mV for the water/DCE system.^28^ The electrochemical cell used had a geometric area of either 0.69 or 1.0 cm^2^ and was similar to that reported elsewhere.15a Optical images of SWCNT interfacial films were recorded with a stereo‐zoom microscope (SMZ168, Motic) connected to a digital live camera (GXCAM‐9, GX Optical). SEM images were obtained by using an FEI XL30 Environmental SEM–FEG operated under high‐vacuum state with an accelerating voltage of 15 keV. XPS was performed by using a K‐Alpha X‐ray photoelectron spectrometer (Fisher scientific) located at the EPSRC NEXUS facility, Newcastle University, UK. The survey spectra were taken at 0.4 eV step size at three different locations on each sample. All experiments were carried out at room temperature.

## Conflict of interest

*The authors declare no conflict of interest*.
